# Tick bite risk factors and prevention measures in an area with emerging Powassan virus disease

**DOI:** 10.1002/puh2.136

**Published:** 2023-12

**Authors:** Nicolette Wilson, Grace M. Vahey, Emily McDonald, Kelly Fitzpatrick, Jennifer Lehman, Sandhya Clark, Kristine Lindell, Daniel M. Pastula, Stephen Perez, Heather Rhodes, Carolyn V. Gould, J. Erin Staples, Stacey W. Martin, Kim Cervantes

**Affiliations:** 1 New Jersey Department of Health, Communicable Disease Service, Trenton, New Jersey, USA; 2 CSTE Applied Epidemiology Fellowship Program, Council of State and Territorial Epidemiologists, Atlanta, Georgia, USA; 3 Epidemic Intelligence Service, Centers for Disease Control and Prevention, Atlanta, Georgia, USA; 4 Arboviral Diseases Branch, Division of Vector-Borne Diseases, National Center for Emerging and Zoonotic Infectious Diseases, CDC, Fort Collins, Colorado, USA

**Keywords:** Powassan virus, prevention, risk factors, tick-borne disease, ticks

## Abstract

**Background:**

In the United States (U.S.), Powassan virus is primarily transmitted to humans by the black-legged tick *(Ixodes scapularis).* Rarely, infections can present as severe neuroinvasive disease. In 2019, four neuroinvasive disease cases were reported in Sussex County, New Jersey, U.S. We administered a survey to county residents to better understand tick bite risk factors and the performance of personal prevention measures.

**Methods:**

A survey was administered in October 2019 to adult residents of randomly selected households. Questions focused on tick bite prevention and risk factors. Crude and adjusted odds ratios (ORs) and 95% confidence intervals were calculated for various outcomes.

**Results:**

Of 274 participants, 25% were previously diagnosed with a tick-borne disease, and 42% reported finding an attached tick in 2019. Yardwork and gardening (OR = 7.38) and spending >50 hours outdoors per week (OR = 8.15) were associated with finding an attached tick. Finding an attached tick was inversely associated with the number of prevention measures used, indicating that a layered approach could reduce the risk of tick bites. Those who performed post-outdoor activity prevention measures (e.g., tick checks) were less likely to have a tick attached compared to finding a crawling tick.

**Conclusion:**

Compliance with prevention recommendations was low, despite a high prevalence of reported tick bites and significant outdoor exposures. Older adults and persons who spend significant time outdoors or engage in yardwork or gardening were at the highest risk of tick bites. Additional research is needed to further understand the barriers to tick bite prevention.

## INTRODUCTION

Powassan virus (POWV) is an RNA virus in the family Flaviviridae that in the United States (U.S.) is most commonly transmitted to humans by the blacklegged tick (*Ixodes scapularis*) [1]. Many human POWV infections are asymptomatic; however, infection can also result in febrile illness or severe neuroinvasive disease, often manifesting as meningitis and encephalitis [2, 3]. In the U.S., most cases of POWV disease are reported in the northeast and Great Lakes regions and usually occur from late spring through mid-fall when blacklegged ticks are most active [2]. During 2009–2018, an average of 15 cases were reported annually in the U.S. with the number of cases reported increasing over time [3, 4].

A variety of factors influence the risk of infection with POWV, including climate, land cover, intermediate hosts and vector ecology, and human behavior [5]. Evidence regarding behaviors or activities that increase the human risk of tick bites varies. Certain demographics (e.g., age and sex), outdoor activities (e.g., hiking, yardwork, and hunting), as well as pet ownership and time spent outside have been associated with an increased risk of tick bites in individual analyses [6-8].

Because there is no specific treatment for, or vaccine to prevent POWV infection, prevention relies on preventing tick bites, particularly as transmission might occur in as little as 15 minuntes from tick attachment [9]. Recommended personal tick bite prevention measures include avoiding tick habitat, using Environmental Protection Agency–registered insect repellents, treating clothing and gear with permethrin, bathing and checking clothing and body for ticks promptly after spending time outdoors [10].

Prior to 2019, eight POWV disease cases were reported in New Jersey (NJ), with the first reported in 2013. The average age of these case-patients was 57 years. Cases were reported in four NJ counties, 63% of them in Sussex County residents [11]. Between May and September 2019, four neuroinvasive POWV disease cases were reported in Sussex County, two of them fatal. All four 2019 cases were male, aged 29–80 years (average 60 years), spent time outdoors in grassy, wooded areas in the local community 30 days before illness onset, and three recalled a recent tick bite. Three of the case-patients lived in two adjacent census tracts, and the fourth lived just outside these census tracts.

In response to this cluster of cases, a previously published investigation was conducted to evaluate POWV seroprevalence and to estimate the proportion of infections leading to neuroinvasive disease in adults in this geographic area [3]. During the investigation, a questionnaire was also administered to all serosurvey participants regarding tick bite prevention and potential exposures. Our aim is to summarize the questionnaire findings to describe the frequency of outdoor activities and tick bite prevention measures among this population and identify potential risk factors for tick bites and groups that might benefit from targeted tick bite prevention messaging to prevent future POWV infections.

## METHODS

### Study design and population

The questionnaire was administered in October 2019 in Sussex County, NJ, U.S., using survey sampling methods previously described in Vahey et al. [3]. All English-speaking adult residents of randomly selected households who lived in the two census tracts where the case cluster occurred since January 1, 2019, were eligible to participate.

### Study variables

Consenting adults were administered a survey (Appendix A) that collected information on demographics, amount of time spent outdoors per week, frequency of specific outdoor activities, whether they had found a tick crawling on or attached to them in 2019, frequency of tick bite prevention measures used prior to (pre-activity prevention) and within two hours after (post-activity prevention) spending time outdoors, pet ownership, and self-reported history of prior tick-borne disease (TBD). Questions about outdoor activities, time spent outside, local recreational areas visited, and tick bite prevention measures were restricted to the 2019 tick season (defined in interviews as “from late spring through fall”).

### Data analysis

Crude odds ratios (ORs) and 95% confidence intervals (CIs) were calculated for outcomes of pre-activity prevention, post-activity prevention, any prevention measures, finding a crawling tick, and finding an attached tick. Adjusted ORs and 95% CIs were calculated using multivariable logistic regression for outcomes of pre-activity prevention, post-activity prevention, any prevention measures, and finding an attached tick. We used stepwise model selection with a stay threshold of 0.1 and starting variables of sex, age group, time spent outdoors, hiking, yardwork/gardening, hunting, birding, running, and picnicking. To determine if there was an association between prevention measures taken this season and a previous TBD diagnosis, we included participants whose TBD diagnosis was greater than one year ago when calculating ORs. Responses for questions regarding the frequency of outdoor activities were categorized into often or sometimes (“frequently”) compared to rarely or never (“infrequently”). Responses to questions regarding tick prevention measures were categorized into always or often (“frequently”) compared to rarely or never (“infrequently”); “sometimes” was removed to create a dichotomous outcome for the analyses. Analyses were performed in SAS (SAS Institute Inc.).

### Ethical considerations

Written consent was obtained from all participants. The Centers for Disease Control and Prevention deemed the project to be non-research public health surveillance, and NJ’s Institutional Review Board approved the project as minimal risk.

## RESULTS

Of 423 randomly selected households, 179 (42%) participated. From the 179 participating households, we enrolled 274 participants; a total of 140 (51%) were female, and the median age was 63 years (IQR, 50–70 years) (Table 1). A quarter (70) of participants had been previously diagnosed with a TBD, and one had been previously diagnosed with POWV disease (Table 1). From January to October 2019, 197 (72%) reported finding a tick crawling on their bodies, and 115 (42%) reported finding an attached tick. Sixty-one percent (168) of participants owned outdoor pets, and more than half of pet owners found crawling or attached ticks on their pets in 2019 (68% and 51%, respectively). Most (57%) participants spent 7–28 hours outdoors per week, whereas 95 (35%) spent more than 29 hours outdoors per week (Table 1).

The most reported outdoor activity was yardwork/gardening (87%; 237). Thirty-four percent (93) of participants reported never using insect repellent when engaging in outdoor activities, and 86% (235) reported never wearing permethrin-treated clothing (Table 2). Fifty-nine percent (162) of participants frequently performed at least one pre-activity tick bite prevention measure; however, no single pre-activity prevention measure was performed frequently by at least half of the participants. Post-activity tick bite prevention measures were performed more often than pre-activity prevention measures. Just under half (130; 48%) of participants reported frequently checking their clothes and 58% (158) checking their bodies for ticks. Tick prevention for pets was used more frequently, with 63% (106) of pet owners reporting they frequently check pets for ticks and 85% (142) reporting use of tick preventive medication on their pets (Table 2).

Females were less likely than males to perform post-activity prevention measures (OR 0.6; 95% CI 0.3–1.0) or to use any prevention measures (OR 0.5, 95% CI 0.3–1.0) (Table 3) but were 2.8 (95% CI 1.3–6.2) times more likely to tuck pants into boots or socks. Males were 2.3 (95% CI 1.4–3.8) times more likely to find an attached tick compared to females; this effect did not change in multivariate analysis.

There was no statistically significant association between age and pre-activity tick bite prevention measures (Table 3). However, participants ≥70 years were less likely (OR 0.3; 95% CI 0.1–0.7) to perform any post-activity prevention measures (Table 3) and to use insect repellent (OR 0.3; 95% CI 0.1–0.8) compared to those <50 years old. Similarly, participants aged 50–69 years were less likely to check their body (OR 0.3; 95% CI 0.1–0.7) or clothes for ticks (OR 0.3; 95% CI 0.2–0.7) than younger age groups. There was no statistically significant association between age and finding an attached tick.

Reporting a prior TBD diagnosis had no effect on the use of pre-activity prevention measures. However, participants with a prior TBD diagnosis were more likely to use post-activity prevention measures (Table 3). Participants with a reported prior TBD diagnosis also had increased odds of finding an attached tick (Table 4).

No variables were significantly associated with performing pre-activity prevention in multivariable analysis. After accounting for sex, time spent outdoors, and all outdoor activities, older age was associated with decreased post-activity prevention measures, with people aged 50–69 years and ≥70 years having ~68% lower odds of performing post-activity prevention (OR 0.3; 95% CI 0.1–0.8 and OR 0.3; 95% CI 0.1–0.8, respectively) compared to younger age groups. People who reported hiking (OR 2.8; 95% CI 1.4–5.6) and doing yardwork/gardening (OR 8.1; 95% CI 3.6–18.7) frequently had significantly higher odds of performing post-activity prevention in multivariable analysis. People who frequently did yardwork/gardening had 3.8 times higher odds (95% CI 1.8–8.3) and people who frequently went hunting had 3.3 times higher odds (95% CI 1.3–9.8) of performing any tick bite prevention measures in multivariable analysis.

Although all post-activity prevention measures were associated with increased odds of finding a crawling or attached tick, the magnitude of the association was larger for finding a crawling tick compared to an attached tick for all post-activity prevention measures except washing/drying clothes (Table 4). Compared to finding a crawling tick, the OR of finding an attached tick for persons reporting showering/bathing soon after returning indoors decreased by 88%. The remaining post-activity prevention measures followed a similar pattern, resulting in a 51%−83% decrease in finding an attached tick. Participants who reported doing yardwork/gardening frequently had the highest odds of finding a crawling (OR 10.1, 95% CI 4.6–22.3) or attached tick (OR 7.4, 95% CI 2.5–21.5) (Table 4) compared to other outdoor activities. Hiking was associated with an increased odds of finding a crawling tick, whereas hunting had an increased odds of finding both a crawling and an attached tick (Table 4). After accounting for effects of age, sex, and all other outdoor activities, doing yardwork/gardening frequently increased the odds of reporting finding an attached tick by 6.2 times (95% CI 2.1–18.2).

Length of time spent outdoors was associated with an increased risk of finding an attached tick. Compared to spending <7 hours per week outdoors, participants who spent 7–28, 29–50, and >50 hours a week outside were 4.6 (95% CI 1.3–16.2), 6.7 (95% CI 1.8–25.1), and 8.2 (95% CI 2.1–31.9) times more likely to find an attached tick, respectively (Table 4).

**FIGURE 1:**
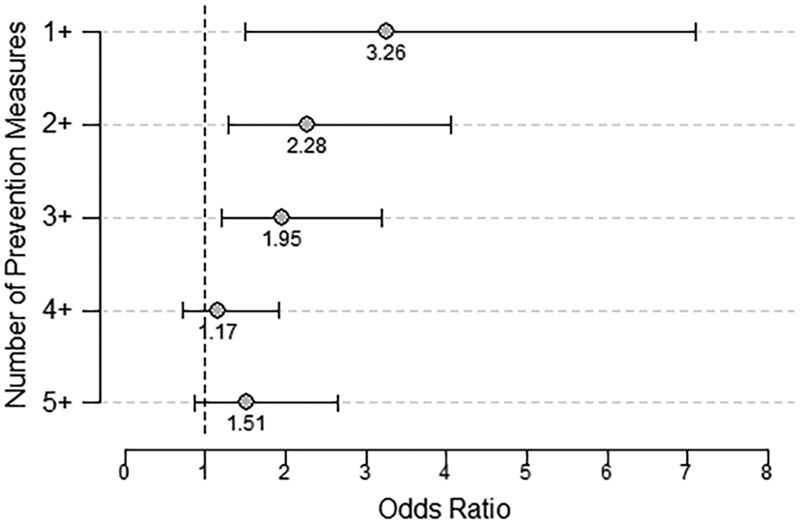
Additive effect of using one or more prevention measures to reduce odds of tick bites. Comparison of the odds ratios of finding a tick attached in relation to the number of prevention measures used. Prevention measures include both pre-and post-activity prevention measures.

Using multiple prevention measures had an additive effect on decreasing the odds of finding an attached tick (Figure 1). Participants using at least one prevention measure had 3.3 times (95% CI 1.5–7.1) higher odds of finding an attached tick compared to those who used none. However, the OR for participants who used two or more prevention measures decreased by 30% and by 54% for those who used five or more.

## DISCUSSION

In this survey of residents in a geographic area where a recent cluster of POWV disease occurred, we found low compliance with recommended tick bite prevention measures, despite a high prevalence of reported tick bites and significant outdoor exposures. We found that only two prevention measures, performing tick checks on themselves and their pets, were performed frequently by more than half of participants. These results are consistent with previous studies on TBD prevention behaviors, which found tick checks were the most used prevention measure [12, 13] and insect repellent was less commonly used [14]. More research aimed at understanding why participants do not frequently perform recommended prevention measures would inform strategies to increase tick bite prevention behavior.

Participants who reported using multiple tick bite prevention measures were less likely to find an attached tick, suggesting a layered approach to prevention could reduce the risk of tick bites. To our knowledge, no previous analyses included the effect of layering prevention measures. Prevention messaging should emphasize a layered approach utilizing both pre- and post-activity prevention measures as performing a single prevention measure is less effective at protecting against tick bites. However, given the overall lack of tick bite prevention in our survey population, suggesting multiple measures may not be effective unless barriers to the use of recommended prevention measures are addressed.

Participants ≥50 years were less likely to use prevention measures after outdoor activities, aligning with previous studies [15, 16]. Older participants were also less likely to use insect repellent prior to spending time outdoors. A meta-analysis by Fischhoff et al. reported the highest incidence of tick bites in children aged five years and under, followed by adults 50–69 years old [17]. Our survey did not include children under the age of 18 but suggests that older adults may be at higher risk for tick bites because of less frequent use of prevention measures. Additionally, when looking at the amount of time spent outdoors, 24% of participants ≥70 years spent ≥29 hours outdoors per week compared to 37% and 40% of participants aged 50–59 and <50 years, respectively. Although older participants spent less time outdoors, this reinforces the need for prevention when spending time outdoors because older adults are still at increased risk even though they spend less time outdoors. Considering the average age of NJ POWV disease case-patients was over the age of 50 years, based on the data reported in the NJ Department of Health (NJDOH) Communicable Disease Surveillance and Reporting System (CDRSS), increased usage of tick bite prevention in the older population is critical.

In our analysis, males were more likely to report doing any tick bite prevention measures but were also more likely to find an attached tick. One potential contributing factor might be that 24% of males in our population spent >50 hours per week outdoors compared to 5% of females. One publication regarding tick prevention behaviors in Delaware found that the proportion of males spending >6 hours per day outdoors was more than double that of females [15]. Although males are more likely to take steps to prevent tick bites, it is possible that they are not performing prevention measures at sufficient intervals related to the length of time spent outdoors. For example, someone may always check for ticks after working outdoors for eight hours, but if tick checks are not performed several times throughout that eight-hour period, ticks will have the opportunity to attach before they can be identified.

As time spent outdoors increased, so did the risk of finding an attached tick. Mead et al. found that the risk of finding an attached tick increased by a factor of 1.1–1.2 for every hour spent outside [7]. Individuals spending significant time outdoors may benefit from targeted tick bite prevention messaging. Additionally, it may be beneficial to suggest using more than one prevention measure and performing tick checks multiple times while spending long periods of time outdoors to further decrease the risk of finding attached ticks.

Yardwork/gardening was the most common outdoor activity reported among participants. Those who reported frequently doing yardwork/gardening were significantly more likely to report finding ticks on themselves. With nearly a quarter of participants reporting not using any prevention measures when engaging in these activities, tick bite prevention messaging should be targeted to this population. Yardwork and gardening usually involve spending long periods of time around the home. According to a previous study in the Northeastern U.S., approximately 86% of the risk of finding ticks on the body was associated with exposures in one’s own yard [7]. Similarly, a New York study found more than 60% of participants with recent tick bites believed they acquired the tick in their own yard [18]. This suggests household level prevention measures (e.g., treating yards) in addition to personal prevention measures may be effective in preventing tick exposures for people who frequently garden and do yardwork.

The application of permethrin to clothing routinely worn during outdoor activities can last up to 3 months and may help address some barriers to compliance with prevention recommendations needing to be applied before or after each outdoor encounter. Eighty-six percent of respondents reported never using permethrin-treated clothes. Low compliance with this recommendation may be due to a combination of lack of knowledge as well as other barriers to use (e.g., not accessible, cost, and safety concerns). For individuals who routinely spend significant time outdoors, determining the barriers to using permethrin-treated clothes is needed.

We found a dramatic decrease in ORs between finding crawling versus attached ticks for almost all post-activity prevention measures. In other words, people who perform prevention measures after outdoor activities reduce their likelihood of a tick bite because they are finding and removing crawling ticks before attachment. This is especially important for POWV disease prevention, as POWV might be transmitted in as little as 15 minutes after attachment [9].

Our study found that outdoor pet ownership was not associated with an increased odds of finding an attached tick. This contrasts with a Canadian study that found dog owners were at a higher risk for tick bites [6]. As noted above, it is theorized that dogs may spend more time in tick habitat and, therefore, expose their owners to ticks. Given our study’s heavily wooded geographic area with abundant peridomestic tick populations, tick habitat might significantly overlap with human space. This would result in participants spending similar amounts of time in tick habitat as outdoor pets and having a similar risk of tick exposure regardless of pet ownership. Additionally, a high percentage of our participants with pets reported using tick prevention frequently on their pets, suggesting that routine pet prevention decreases any additional risk of tick bites related to pet ownership. Niesobecki et al. found tick prevention for pets was the most frequently used prevention measure in endemic areas and attributed this to the perception that pets spend more time in tick habitat, such as wooded areas that contain leaf litter, and therefore, pet owners perceive their pets’ risk for tick bites to be higher than their own [13]. Additionally, pet tick prevention often requires monthly application compared to before, during, and after every outdoor activity for human prevention measures, suggesting that the frequency of tick bite prevention could be a barrier to its use.

Our study is subject to several limitations. First, our results are biased toward older adults as most of the participants were over the age of 60. Therefore, these results are not generalizable to areas outside of our study population with different demographic characteristics. Our questionnaire asked for specific information from the previous 6–9 months, such as the number of crawling or attached ticks, the frequency of outdoor activities, and tick bite prevention measures, all of which are subject to recall bias. POWV disease cases are often reported through November. By conducting our interviews in October, prior to the end of tick season, we might have failed to capture additional tick exposures and activities related to the risk of POWV infection. Additionally, the number of tick encounters may be underestimated by participants because the black-legged tick’s nymphal stage, which predominates in the spring and summer [19], can easily be missed, especially by older adults. Prior TBD diagnosis was self-reported and not confirmed with medical record review but still measures whether a participant believes they had a TBD and how that impacted their likelihood of performing prevention measures. Our survey did not ask questions regarding barriers to performing prevention measures, which would aid in developing improved prevention guidance for this population. Additionally, our survey did not assess how correctly or thoroughly participants implemented tick bite prevention measures, and differences may impact the likelihood of tick bites and could confound results. Finally, our survey focused on personal prevention measures, but tick bite prevention also occurs at household (e.g., landscaping and yard insect treatments) and community levels (e.g., deer population control). Our survey did not include questions regarding household prevention measures that may have affected respondents’ overall risk of tick exposures.

## CONCLUSION

Although POWV disease is uncommon, the increased incidence in this geographic area of NJ and associated severe outcomes reinforce the need for ongoing diligence regarding tick bite prevention and community-wide tick bite prevention messaging. Messaging should be targeted to the highest risk groups, including older adults and persons who spend significant time outdoors or engage in yardwork or gardening. Educational campaigns should emphasize that using a layered approach consisting of multiple prevention measures is likely to have the greatest impact on reducing the risk of tick bites and TBD and that pre-activity prevention measures are effective when performed correctly. In our survey population, tick bite prevention was most often performed after coming indoors, which may be less effective at preventing POWV infection because of the short attachment time required for POWV transmission and for persons who spend a significant number of hours outdoors at one time. In addition to personal prevention measures, household-level prevention should be recommended as a method to reduce tick populations in individuals’ yards. Future studies to provide additional insight on barriers to prevention use are needed to design the most effective prevention messaging and interventions. With increased awareness of POWV disease, the public might be more receptive to adopting practices for protecting themselves and their loved ones from POWV.

## Supplementary Material

Appendix A: Participant questionnaire on tick bite risk factors and prevention behaviors– Sussex County, New Jersey, 2019

## Figures and Tables

**TABLE 1:** Participant demographics and risk factors for tick exposures—Sussex County, New Jersey, 2019 (*N* = 274).

	Characteristics	
	*n*	%
**Median age (years)**	63.0 (IQR, 50–70)	
**Age group**		
<50	67	24.5
50–69	138	50.4
≥70	69	25.2
**Sex**		
Male	135	49.3
Female	139	50.7
**Retired from work**		
Yes	103	39.8
**Year-round residence**		
Yes	264	96.4
**Diagnosed with a tick-borne disease**		
Yes	70	25.0
**Diagnosed with Powassan virus disease**		
Yes	1	0.3
**Median residency duration (years)**	20.0 (IQR, 9–33)	
**Risk factors**		
**Pet owners**		
Yes	168	61.3
**Found tick crawling pet in 2019** ^†^		
Yes	115	68.5
**Found tick attached pet in 2019** ^†^		
Yes	86	51.2
**Found tick crawling in 2019**		
Yes	197	71.9
**Frequency of ticks crawling**		
1–2	80	40.6
3–10	87	44.2
10+	30	15.2
**Found tick attached in 2019**		
Yes	115	42.0
**Frequency of ticks attached**		
1–2	90	78.3
3–10	22	19.1
10+	3	2.6
**Time spent outdoors (per week)**		
< 7 hours	23	8.4
7–28 hours	155	56.6
29–50 hours	55	20.1
>50 hours	40	14.6
Unknown	1	0.3

† *N* = 168.

**TABLE 2:** Participant tick exposure activities and frequency of tick bite prevention measures used—Sussex County, New Jersey, 2019(*N* = 274).

	Frequency *n* (%)				
	Never	Rarely	Sometimes	Often	Always
**Exposure activity**					
Hiking	116 (42.3)	60 (22.0)	56 (20.4)	42 (15.3)	
Yardwork/gardening	19 (6.9)	18 (6.6)	59 (21.5)	178 (65.0)	
Hunting	171 (62.4)	33 (12.0)	40 (14.6)	30 (11.0)	
Birding	210 (76.6)	30 (11.0)	20 (7.3)	14 (5.1)	
Running/walking	46 (16.8)	43 (15.7)	80 (29.2)	105 (38.3)	
Picnic (dining outside)	26 (9.5)	29 (10.6)	120 (43.8)	99 (36.1)	
**Pre-activity prevention**					
Repellent on skin	93 (33.9)	68 (24.8)	65 (23.7)	31 (11.3)	17 (6.2)
Wear repellent-treated clothes	235 (85.8)	13 (4.7)	12 (4.4)	7 (2.55)	7 (2.55)
Wear long sleeves	63 (23.0)	49 (17.9)	88 (32.1)	47 (17.2)	27 (9.9)
Wear long pants	69 (10.6)	27 (9.9)	86 (31.4)	56 (20.4)	76 (27.7)
Tuck pants into socks/boots	189 (69.0)	22 (8.0)	30 (11.0)	12 (4.4)	21 (7.7)
**Post-activity prevention**					
Check clothes for ticks	63 (23.0)	28 (10.2)	53 (19.3)	58 (21.2)	72 (26.3)
Check body for ticks	30 (11.0)	26 (9.5)	60 (21.9)	67 (24.5)	91 (33.2)
Another person checks for ticks	106 (38.7)	37 (13.5)	67 (24.5)	37 (13.5)	27 (9.9)
Bathe immediately after	57 (20.8)	32 (11.7)	74 (27.0)	61 (22.3)	50 (18.3)
Wash/dry clothes	129 (47.1)	43 (15.7)	45 (16.4)	33 (12.0)	24 (8.8)
Check pets for ticks^†^	13 (7.7)	19 (11.3)	30 (17.9)	55 (32.7)	51 (30.4)
Use tick prevention on pets^†^^‡^	15 (8.9)	2 (1.2)	6 (3.6)	36 (21.4)	106 (63.1)

† *N* = 168

‡ 3 responses were N/A.

**TABLE 3:** Tick prevention measures use stratified by age, sex, and prior tick-borne disease diagnosis—Sussex County, New Jersey, 2019 (*N* = 274).

Prevention measures	Any pre-activity prevention				Any post-activity prevention				Any prevention			
	OR	95% CI		*p*	OR	95% CI		*p*	OR	95% CI		*p*
**Sex**												
Male^†^												
Female	**1.2**	0.8	2.0	0.461	**0.6**	0.3	1.0	0.059	**0.5**	0.3	1.0	0.047
**Age**												
<50^†^												
50–69	**0.8**	0.5	1.5	0.547	**0.4**	0.2	0.9	0.025	**0.4**	0.2	1.1	0.099
≥70	**0.9**	0.4	1.7	0.728	**0.3**	0.1	0.7	0.006	**0.5**	0.2	1.3	0.206
**Tick-borne disease** ^‡^												
No^†^												
Yes	**1.1**	0.6	1.9	0.883	**3.75**	1.6	8.7	0.001	**2.4**	0.9	6.4	0.105

*Note*: Excluding individuals diagnosed with a tick-borne disease within the year prior to interview date.

Abbreviations: CI, confidence interval; OR, odds ratio.

† Reference.

‡ *N* = 61.

**TABLE 4:** Odds ratio of finding a tick crawling or attached by select prevention measuresˆ and risk factors—Sussex County, New Jersey, 2019 (*N* = 274).

Prevention measure	Tick crawling				Tick attached				Percent change^¶^
	OR	CI		*p*	OR	CI		*p*	
Check body for ticks	**19.7**	7.0	55.2	<0.0001	**9.6**	2.7	34.0	<0.0001	−51%
Check clothes	**6.9**	3.1	15.4	<0.0001	**3.0**	1.5	6.3	0.003	−56%
Another person check	**21.1**	2.8	161.1	<0.0001	**3.6**	1.5	8.6	0.01	−83%
Shower/bathe	**28.6**	6.3	129.2	<0.0001	**3.6**	1.6	8.0	0.003	−88%
Wash/dry clothes	**2.4**	0.8	6.7	0.11	**1.1**	0.4	2.6	1.00	−55%
**Exposure/risk factors**									
Hiking	**2.7**	1.5	5.1	0.001	**1.5**	0.9	2.4	0.16	−46%
Yardwork/gardening	**10.1**	4.6	22.3	<0.0001	**7.4**	2.5	21.5	<0.0001	−27%
Hunting	**2.6**	1.3	5.2	0.01	**2.5**	1.4	4.3	0.002	−5%
Birding	**2.5**	0.9	6.7	0.07	**1.5**	0.7	3.2	0.27	−39%
Running/walking	**1.1**	0.6	1.9	0.78	**1.4**	0.8	2.3	0.24	27%
Picnic	**1.5**	0.8	2.8	0.24	**1.5**	0.8	2.8	0.22	3%
Pet ownership^‡^	**1.5**	0.9	2.5	0.17	**1.1**	0.7	1.8	0.71	−24%
Tick-borne disease diagnosis^§^	**0.4**	0.2	0.8	0.006	**1.8**	1.0	3.2	0.054	350%
**Time spent outdoors** (per week)									
<7 hours^†^									
7–28 hours	**1.4**	0.6	3.4	0.48	**4.6**	1.3	16.2	0.01	232%
29–50 hours	**1.9**	0.7	5.3	0.28	**6.7**	1.8	25.1	0.002	254%
>50 hours	**4.5**	1.3	15.8	0.03	**8.2**	2.1	31.9	0.001	81%

Abbreviations: CI, confidence interval; OR, odds ratio.

† Reference.

‡ *N* = 168.

§ *N* = 61 Excluding individuals diagnosed with a tick-borne disease within the year prior to interview date.

¶ Percent change when comparing odds of finding a crawling tick to odds of finding an attached tick.

ˆ Pre-prevention measures not included because of lack of significance.

## Data Availability

The data that support the findings of this study are available from the corresponding author upon reasonable request.
