# Supplementary material for: Tick bite risk factors and prevention measures in an area with emerging Powassan virus disease
Source: Public Health Chall. Author manuscript; Available in PMC 2024 May 24. (PMC11118757; doi:10.1002/puh2.136)
Supplement: Appendix A: Participant questionnaire on tick bite risk factors and prevention behaviors– Sussex County, New Jersey, 2019 [file NIHMS1989249-supplement-Appendix_A__Participant_questionnaire_on_tick_bite_risk_factors_and_prevention_behaviors__Sussex_County__New_Jersey__2019.pdf]

Appendix A: Participant questionnaire on tick bite risk factors and prevention behaviors– Sussex County, New Jersey, 2019

Interviewer: ____________________________

ID Sticker:

Date of interview: ______/______/_________

Demographic information

1. What is your age? _________ years

2. What is your sex?
☐ Male ☐ Female ☐ Other ☐ Prefer not to answer

3. What is your occupation?
____

4. How long have you lived at your current address?
________________ ☐ Months ☐ Years

5. Is this your residence year-round? ☐ Yes ☐ No
a. If no, where is your other residence? ____
b. If no, what months did you spend in Sussex County this year?
____

Exposure information

6. During this year's tick season, which lasts from late spring through fall, how much time did you spend outdoors each week?
Including your backyard, parks, trails, etc. for personal and work reasons.
☐ <7 hours *(<1 hour per day)* ☐ 7-28 hours *(1-4 hours per day)* ☐ 29-50 hours *(5-7 hours per day)* ☐ >50 hours *(>7 hours per day)*

7. During this year's tick season, which lasts from late spring through fall, about how often did you engage in the following outdoor activities?

	Never	Rarely	Sometimes	Often
a. Hiking/camping	☐	☐	☐	☐
b. Gardening/yardwork/mowing	☐	☐	☐	☐
c. Hunting/fishing	☐	☐	☐	☐
d. Birding/bird watching	☐	☐	☐	☐
e. Running/walking	☐	☐	☐	☐
f. Picnic/barbecue/etc	☐	☐	☐	☐
g. Other activity (specify): _________________________	☐	☐	☐	☐

8. During this year's tick season, which lasts from late spring through fall, how often did you do each of the following before you spent time outdoors?					
	Never	Rarely	Sometimes	Often	Always
a. Apply insect repellent to skin?	☐	☐	☐	☐	☐
b. Wear clothes treated with an insect repellent such as permethrin?	☐	☐	☐	☐	☐
c. Wear long sleeves?	☐	☐	☐	☐	☐
d. Wear long pants?	☐	☐	☐	☐	☐
e. Tuck pants into socks or boots?	☐	☐	☐	☐	☐

9. During this year's tick season, which lasts from late spring through fall, how often did you do each of the following immediately after spending time outdoors?					
	Never	Rarely	Sometimes	Often	Always
a. Check your clothes for ticks?	☐	☐	☐	☐	☐
b. Check your body for ticks?	☐	☐	☐	☐	☐
c. Have another person check you for ticks?	☐	☐	☐	☐	☐
d. Bathe or shower?	☐	☐	☐	☐	☐
e. Wash/dry or dry clothing?	☐	☐	☐	☐	☐
f. Check your pets for ticks? ☐ *Check here if no outdoor pets.*	☐	☐	☐	☐	☐

10. During which activities did you use *any* tick prevention measures? *Check all that apply*

☐ Hiking/camping ☐ Gardening/yardwork/mowing ☐ Hunting/fishing
☐ Birding/bird watching ☐ Running/walking ☐ Picnic/BBQ/etc
☐ Other activity (specify): ________________________________ ☐ None

11. During this year's tick season, which lasts from late spring through fall, about how many times did you visit the following recreational areas in Sussex County?
Don't include visits where you were inside a car/other enclosed vehicle the entire time.

a. Bear Swamp Wildlife Management Area __________
b. Stokes State Forest __________
c. Delaware Water Gap National Recreation Area __________
d. Swartswood State Park __________
e. Hampton Township Recreational Area __________
f. Frankford Park __________
g. High Point State Park __________

12. Since January 1, 2019, do you recall any of the following?

a. Finding a tick crawling on your body (skin or clothing) ☐ Yes ☐ No ☐ Unknown
b. If yes, how many times? ☐ 1-2 times ☐ 3-10 times ☐ More than 10 times
c. Finding a tick attached to your body ☐ Yes ☐ No ☐ Unknown
d. If yes, how many times? ☐ 1-2 times ☐ 3-10 times ☐ More than 10 times

13. Do you have any household pets that spend any time outdoors?
☐ Yes ☐ No

14. If yes, since January 1, 2019, do you recall any of the following?
a. Finding a tick crawling on your pet ☐ Yes ☐ No ☐ No pets
b. Finding a tick attached to your pet ☐ Yes ☐ No ☐ No pets

15. Do your outdoor pet(s) receive tick prevention (e.g. through a tick collar, monthly spot-on treatment)
☐ N/A ☐ Never ☐ Rarely ☐ Sometimes ☐ Often ☐ Always

16. Have you been vaccinated for any of the following?
☐ Yes ☐ No ☐ Unknown Yellow fever If yes, about what year? ___________
☐ Yes ☐ No ☐ Unknown Japanese encephalitis If yes, about what year? ___________
☐ Yes ☐ No ☐ Unknown Tick-borne encephalitis If yes, about what year? ___________

Illness information

17. Have you ever been diagnosed with a Powassan virus infection?
☐ Yes ☐ No ☐ Don't know

a. If yes, when? (MM/YYYY) ______/________________

b. If yes, were you hospitalized? ☐ Yes ☐ No

18. Have you been hospitalized for any reason besides a planned surgery in the past three years?
☐ Yes ☐ No

a. If yes, were you hospitalized for encephalitis or meningitis?
☐ Yes ☐ No ☐ Not Sure
If you are unsure, describe why you were hospitalized:
____
____

19. Have you ever been diagnosed by a health care provider as having any other tickborne disease (such as Lyme disease, anaplasmosis, babesiosis, ehrlichiosis, or Rocky Mountain spotted fever)? If yes, when?
In the past year ☐ Yes ☐ No
1-5 years ago ☐ Yes ☐ No
More than 5 years ago ☐ Yes ☐ No
